# Prospective study of peripartum group B streptococcus colonization in Japanese mothers and neonates

**DOI:** 10.1017/S0950268824001560

**Published:** 2025-01-06

**Authors:** Emiko Yoshida, Jun Takeda, Yojiro Maruyama, Naoko Suga, Satoru Takeda, Hajime Arai, Atsuo Itakura, Shintaro Makino

**Affiliations:** 1 Department of Obstetrics and Gynecology, Juntendo University, Tokyo, Japan; 2 Diagnostics and Therapeutics of Intractable Diseases, Intractable Disease Research Center, Graduate School of Medicine, Juntendo University, Tokyo, Japan; 3 Department of Obstetrics and Gynecology, Juntendo University Nerima Hospital, Tokyo, Japan; 4 Department of Obstetrics and Gynecology, Juntendo University Urayasu Hospital, Chiba, Japan; 5 ChatGPTHe is the Director of the Aiiku Research Institute for Maternal, Child Health, and Welfare, Tokyo, Japan; 6 Department of Neurosurgery, Juntendo University, Tokyo, Japan

**Keywords:** group B streptococcus, vertical transmission, capsular types, *Streptococcus agalactiae*, real-time polymerase chain reaction

## Abstract

Group B streptococcus (GBS) is a major global cause of neonatal, infant, and maternal infections. In Japan, national guidelines based on Centers for Disease Control and Prevention recommendations mandate culture-based screening and intrapartum antibiotic prophylaxis (IAP) for GBS-positive pregnant women. Despite initial reductions in GBS infections, the incidence has plateaued, and there are notable limitations in current prevention methods. Approximately 15% of pregnant women are not screened for GBS, and intermittent colonization undermines screening accuracy, contributing to early-onset disease. IAP does not prevent late-onset disease, the incidence of which is increasing in Japan. This study reviewed maternal and neonatal GBS colonization using polymerase chain reaction, evaluated capsular type distributions, and explored late-onset disease infection routes. Among 525 mother-neonate pairs, the study found a higher detection rate of GBS via polymerase chain reaction compared to culture methods and identified significant discrepancies between antepartum and intrapartum colonization. GBS was detected in 3.5% of neonates from initially negative mothers at 4 days of age. Capsular types varied between mothers and neonates, indicating potential horizontal transmission. This study underscores the need for improved rapid diagnostic tests and highlights the potential of maternal GBS vaccination as a future prevention strategy.

## Key points

- PCR testing at the time of delivery showed higher sensitivity and specificity for GBS than screening during pregnancy.
- Approximately 60% of the neonates born to GBS-positive mothers were colonized with GBS within 4 days postpartum.
- Approximately 5.7% of neonates from GBS-negative mothers at delivery were colonized with GBS within 1 month of birth.
- Differences in the capsular types of GBS isolates from mothers and neonates suggest potential horizontal transmission.
- These findings highlight the effectiveness of PCR testing at the time of delivery, the issues surrounding GBS transmission dynamics, and advancements in vaccine development.

## Introduction

Group B streptococcus (GBS) is an important cause of disease worldwide, contributing to neonatal and infant infections, deaths, disabilities, stillbirths, and maternal infections.

National guidelines for the prevention of vertical GBS transmission in Japanese perinatal care were issued in 2008, following the latest 2020 guidelines of the Centers for Disease Control and Prevention (CDC), which recommend culture-based universal screening and intrapartum antibiotic prophylaxis (IAP) for pregnant women colonized with GBS during labour [1]. A significant reduction in the incidence of GBS infections has been reported since the implementation of IAP policies; the decreasing trend has now stagnated, and the incidence has stabilized. The reasons for persistent GBS infection are as follows. First, 15% of pregnant women are not tested for GBS antepartum due to reasons such as preterm delivery [2, 3]. Second, there was a poor correlation between antenatal screening results and intrapartum maternal GBS colonization due to intermittent vaginal colonization [4–7]. In fact, 80% of early-onset (EOD; birth to 6 days) cases are infants born to mothers with unknown or negative antepartum GBS [4, 7–9]. Molecular tests that allow rapid testing at the time of labour with high diagnostic sensitivity and specificity are useful to optimize IAP.

Third, IAP has no impact on the prevention of late-onset disease (LOD: 7–89 days) [2]. In fact, a national survey conducted in Japan from 2016 to 2020 [10] reported a significant increase in the total incidence of GBS infections over 5 years, mainly due to an increase in LOD. Currently, GBS is the most common cause of paediatric bacterial meningitis in Japan [11, 12]. The epidemiology of GBS varies considerably both geographically and over time. The pathogenesis of LOD is less well understood than that of EOD, and the infection route in some infants remains unclear [13–17]. As a preventive measure for LOD, several capsular serotype-specific GBS vaccines have recently been developed [18]. In 2015, the World Health Organization (WHO) prioritized the development of GBS vaccines suitable for maternal immunization during pregnancy and their use in low- and middle-income countries. GBS vaccine candidates are currently being evaluated in clinical trials, and positive outcomes are anticipated [19]. Cost-effectiveness analyses of maternal vaccination against GBS in Japan have shown it is economically viable [20]. Therefore, detailed epidemiological data on the maternal and neonatal burden and capsular types are crucial for decision-making regarding interventions in perinatal care but are currently lacking.

In this study, we attempted to review the clinical epidemiology of colonizing GBS in pregnant women and neonates using PCR, describe the GBS capsular type distribution in infants and those colonizing pregnant women for risk assessment and future vaccination strategies, and discuss the unresolved infection routes of LOD.

## Methods

### Sample collection

Women who underwent pregnancy checkups after 34 weeks of gestation and delivered at any of the three Juntendo University hospitals between September 2022 and March 2023 were included. Up to 10 swabs were taken, consisting of four swabs from women (samples A, B, C, and D) and six swabs from their neonates (samples P, Q, R, S, T, and U). Sample A (antepartum) included vaginal and rectal samples taken at the same time as the GBS screening culture swab after 34 weeks (approximately at 36 weeks) in the third trimester. Sample B (labour onset) was collected from the vagina and rectum at the time of admission for labour, and sample C (postpartum) was collected from the vagina and rectum 1 day before discharge. All vaginal and rectal swabs were collected according to the recommendations of the CDC.

Sample D (breast milk) was provided only by GBS-positive women, as defined in the Guidelines for the Detection and Identification of Group B Streptococcus (American Society for Microbiology Clinical and Public Health Microbiology Committee) [21] and the Guidelines for Obstetrical Practice (Japan Society of Obstetrics and Gynecology and Japan Association of Obstetricians and Gynecologists) [1]. Sample D was collected by absorbing breast milk with a swab at approximately 1 month postpartum at the outpatient clinic. Samples P, R, and T were collected from the neonates’ oral cavity, and samples Q, S, and U were collected from the neonatal rectum. Samples P (day 0; oral) and Q (day 0; rectal) were collected immediately after birth. Samples R (day 4, oral) and S (day 4, rectal) were collected 1 day before discharge. Samples T (day 30, oral) and U (day 30, rectal) were collected at approximately 1 month of age at the outpatient clinic.

All swab samples included in the study were stored at −80°C within 6 h of sample collection until nucleic acid extraction. Clinical signs and symptoms of infection were observed in all neonates until the end of the third month.

The participants completed self-administered medical questionnaires on family composition, infertility treatment, medication for the reproductive cycle, and experience with the GBS culture test.

### GBS culture

Vaginal-rectal swabs were plated directly on CHROMagar™ StrepB (KANTO CHEMICAL, Tokyo Japan) and incubated under aerobic conditions at 35 ± 1 °C. After 24 and 48 h, the plates were checked for growth of GBS with mauve colonies on chromogenic media, no growth, or other colour growth. Mauve colonies were subcultured on 5% sheep blood agar (BBL, BD Diagnostics, Sparks, MD) for 18–48 h. Presumptive identification of isolated colonies was made using latex agglutination (Seroiden Strepto Kit ‘EIKEN,’ Eiken Chemical, Tokyo, Japan) in Nerima Hospital, while the latest Bruker Biotyper matrix-assisted laser desorption ionization-time of flight mass spectrometry system (BD Diagnostic Systems, Sparks, MD) was used at the Juntendo University Hospital and Urayasu Hospital. GBS identification was confirmed by morphology, haemolytic activity on sheep blood agar plates, biochemical reactions, or a combination of these tests.

### DNA purification

The swab specimens from pregnant women and neonates were suspended with 800 μl of the suspension buffer (10 mM MES, pH 5.8, 1 mM MgCl_2_). To disrupt the capsule of gram-positive bacteria, 4.8 units of mutanolysin (Sigma-Aldrich, Cat# M9901) were added to 240 μl of the suspended sample, and the mixture was incubated at 37°C for 10 min. After treatment with mutanolysin, the sample was mixed with 480 μl of 1.5 × SSB (DNAFORM, Yokohama, Japan). DNA purification from the sample was performed using the SmartExtract kit (DNAFORM) according to manufacturer instructions. The DNA from the spin column was eluted with 120 μl of eluting solution.

### Polymerase chain reaction

GBS was detected in the isolated DNA sample by polymerase chain reaction (PCR) using a GBS detection and capsule typing kit (DNAFORM). The PCR was carried out with a real-time PCR instrument, CFX Opus 96 (Bio-Rad Laboratories, Hercules, CA), under the following thermal conditions: 95°C for 2 min, 50 cycles of two-step thermal conditions at 95°C for 10 s and 60°C for 30 s, followed by a thermal step at 30°C for 5 s. The fluorescence signal was detected using a FAM filter after the second thermal step at 60°C for 30 s for each cycle.

### Statistical analysis

We collected and confirmed research data using REDCap, an electronic data capture system hosted at Juntendo University [22]. Access to servers and systems is restricted by user accounts and passwords, and a full audit trail is recorded.

Univariate analysis was used to identify risk factors for positive GBS test results based on antepartum culture screening during the latest pregnancy. For the multivariate analysis, odds ratios using conditional logistic regression were used to adjust for variables with *p*-values <0.001 detected with the univariate analysis. For the identification of risk factors for positive GBS test results based on antepartum culture screening during the latest pregnancy, we also included maternal age, parity, household composition, opportunities for contact with infants within the last year, and history of use of menstrual control drugs. Data was analyzed using SPSS Statistics software version 29.0.2.0 (20) (IBM, New York, USA).

### Ethical considerations

This study was approved by the Research Ethics Committee of the Faculty of Medicine, Juntendo University (number: H20-0247). The authors assert that all procedures contributing to this work comply with the ethical standards of the relevant national and institutional committees on human experimentation and with the Helsinki Declaration of 1975, as revised in 2008. All participants provided written informed consent prior to enrolment.

## Results

### Maternal characteristics and obstetric data

**Figure 1.**
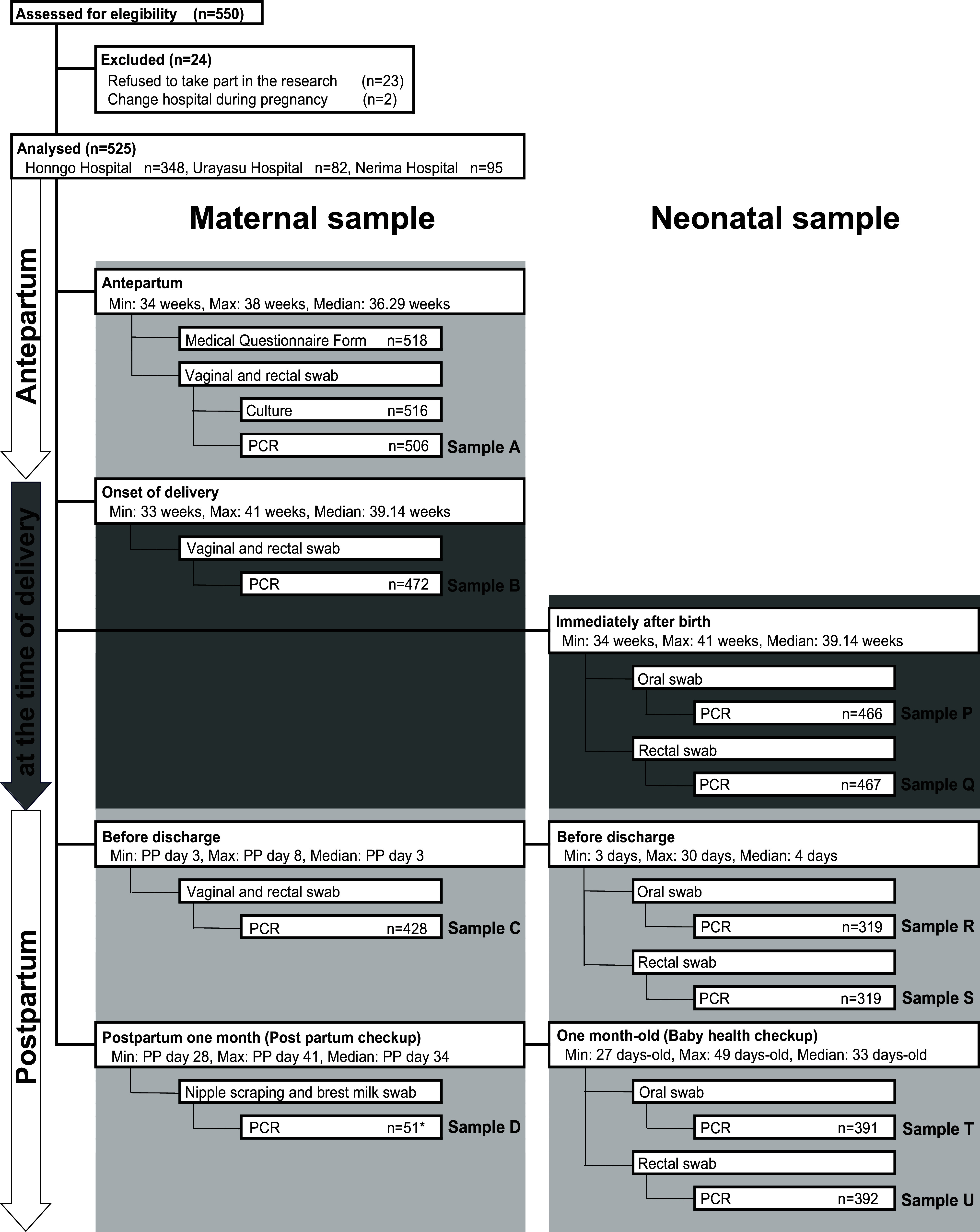
Study design, sample collection process and GBS detection methods from the antepartum to postnatal stages. The maternal swabs were collected from the vagina and rectum, and neonatal swabs were collected from the oral cavity or rectum. Sample D was collected from breast milk of GBS positive women with scraping nipples by swabs (*). For culture testing, GBS selective culture medium were used. For PCR testing, DNA amplification was performed without an enrichment cultivation.

This prospective study was conducted at Juntendo University Hospital, Tokyo; Juntendo University Urayasu Hospital (Urayasu), Chiba; and Juntendo Nerima Hospital (Nerima), Tokyo. Each hospital has been designated as a Perinatal Medical Center by the Tokyo Metropolitan Government or the Chiba Prefecture Government. Among the 550 enrolled women, 25 were excluded: 23 refused to participate, and two changed hospitals before delivery (Figure 1). The final sample consisted of 525 cases.

The maternal characteristics and delivery outcomes are presented in Tables 1 and 2, respectively. The median [percentiles 25th–75th] maternal age was 35 [30–37] years, and 63.2% of the women were nulliparous (Table 1). Approximately 60% of participants spontaneously conceived (Table 1). The results of the antepartum GBS screening by culture included 82.1% negative and 17.0% positive results. Only 0.9% of participants could not undergo GBS screening by culture before delivery.

**Table 1.** Maternal characteristics and obstetric data

Variable	Total *n* = 525
Age (years), median [P25–75]	35 [31–38]
Gravidity, median [P25–75]	2 [1–2]
1, *n* (%)	247 (47.0)
2, *n* (%)	189 (36.0)
3 or more, *n* (%)	89 (17.0)
Parity, *n* (%)	0 [0–1]
0, *n* (%)	332 (63.2)
1, *n* (%)	162 (30.9)
2, *n* (%)	26 (5.0)
3 or more, *n* (%)	5 (1.0)
Penicillin allergy, *n* (%)	11 (2.1)
Other antibacterial allergy, *n* (%)*	17 (3.2)
Infertility treatment, *n* (%)	201 (38.3)
Timed intercourse	39 (7.4)
Artificial insemination	11 (2.1)
Embryonic transplantation	150 (28.6)
Egg donation	1 (0.2)
Results of antepartum GBS screening by culture	
Positive at least once, *n* (%)	89 (17.0)
Negative, *n* (%)	431 (82.1)
Never examined until delivery	5 (0.9)

*Note*: P25–75: interquartile range [percentiles 25th–75th].

* : One sulfa drugs, six cephems, four macrolides, three tetracyclines, six new quinolones (with overlap).

**Table 2.** Delivery outcomes

Variable	Total *n* = 525
Gestational age at birth (weeks), median [P25–75]	39 [38–40]
Gestational age < 36 weeks, *n* (%)	8 (3.2)
Premature rupture of the membranes (PROM), *n* (%)	137 (26.1)
Rupture of the membranes at the time of admission, *n* (%)	121 (23.0)
Delivery method	
Vaginal, *n* (%)	378 (71.8)
Cesarean (elective), *n* (%)	68 (13.0)
Cesarean (emergency), *n* (%)	80 (15.2)
Reason of emergency cesarean	
Non-reassuring foetal status (except chorioamnionitis), *n* (%)	33 (6.3)
Obstructed labour, *n* (%)	12 (2.3)
Preterm onset of labour / PROM, *n* (%)	0 (0.0)
Chorioamnionitis, *n* (%)	3 (0.6)
Others, *n* (%)	32 (6.0)
Neonatal intensive care unit (NICU) admission, *n* (%)	22 (4.2)
Neonatal GBS infection, *n* (%)	0 (0.0)

*Note*: P25–75: interquartile range [percentiles 25th–75th].

Considering the delivery outcomes (Table 2), preterm delivery (gestational age < 36 weeks) only occurred in 3.2% of cases. A total of 26.1% of labours began with premature rupture of membranes (PROM), and 88% of these PROM cases had ruptured membranes at the time of admission. Most cases involved vaginal delivery (71.8%). The indications for the 80 cases of emergency caesarean section included non-reassuring fetal status (33/80 = 41.3%), obstructed labor (12/80 = 15.0%), and chorioamnionitis (3/80 = 3.8%). Twenty-two neonates (4.2%) were admitted to the Neonatal Intensive Care Unit, but there were no cases of neonatal GBS infection.

### Risk factors for positive maternal GBS test results based on antepartum culture screening in the latest pregnancy

Participants provided information on each risk factor by completing self-administered medical questionnaires before antepartum GBS culture screening. The rate of valid responses to the self-administered medical questionnaires was 98.7% (Supplementary Table S1). Odds ratios (OR) were calculated for each factor. Age was classified into 2 groups: <35 years and ≥ 35 years. This cutoff was based on the median participant age of 35 years. A history of positive culture tests for GBS was a risk factor for a positive antepartum culture screening test for GBS during the latest pregnancy in both the univariate (OR: 5.316; 95% confidence interval: 2.422–11.671) and multivariate (OR: 5.270; 95% confidence interval: 2.330–11.917) analyses (Table 3). Although living with infants was more common in participants with positive culture-based antepartum GBS screening test results than living with only adults (>18 years of age), no increased risk was found in the analysis. Similarly, there was no significant difference in the risk of GBS colonization between primiparas and multiparas (Table 3).

**Table 3.** Comparison of features between GBS-positive and GBS-negative pregnant women based on antepartum GBS culture screening in the latest pregnancy

		GBS positive test rate^*1^	Univariate-Odds Ratio(95% CI)	Multivariate-Odds Ratio (95% CI)^*2^
Age	<35 years old (*n* = 252)	15.9% (40/252)	0.991	0.998
	≤35 years old (*n* = 254)	15.7% (40/254)	(0.614–1.597)	(0.606–1.641)
Primiparous/multiparous	Multiparous (*n* = 184)	20.7% (32/184)	1.202	0.512
	Primiparous (*n* = 322)	14.9% (48/322)	(0.737–1.960)	(0.741–5.857)
Family living together	With infants (with/without 7–18-year-old children) (*n* = 131)	20.6% (27/131)	1.577	2.676
	With over 18-year-old adults only (*n* = 372)	14.2% (53/372)	(0.944–2.636)	(0.830–8.634)
Opportunities for contact with infantswithin the last year	Yes (*n* = 206)	18.0% (37/206)	1.309	0.928
	Never (*n* = 300)	14.3% (43/300)	(0.809–2.116)	(0.449–1.918)
History of menstrual control drug use	Yes (*n* = 230)	17.7% (41/230)	1.318	1.208
	Never experience (*n* = 276)	14.1% (39/276)	(0.817–2.127)	(0.736–1.981)
Previous positive GBS culture tests^*3^	Yes (*n* = 28)	46.4% (13/28)	5.316	5.27
	Never (*n =* 478)	14.0% (67/478)	(2.422–11.671)	(2.330–11.917)

*Note*:

*1 GBS positive rate of antepartum GBS culture screening

*2 Multivariate-odds ratio (95% CI)-calculated odds ratio with 95% confidence of disease using logistic regression.

*3 It is based on self-reported data of the results of culture tests carried out in the past, regardless of pregnancy or non-pregnancy.

CI, confidence interval.

### Fluctuations in maternal GBS colonization during pregnancy and comparison of GBS detection rates in culture and PCR tests

The rates of antepartum GBS colonization detected by culture and PCR were 16.1% (83/516) and 18.6% (94/505), respectively, at a gestational age of 36.29 weeks (Table 4). The rates of GBS colonization at delivery onset detected by culture and PCR were 11.7% (11/94) and 16.6% (78/470), respectively, at a median gestational age of 39.14 weeks. The detection rate of PCR was superior to that of culture at both measurement points.

**Table 4.** Results of antepartum GBS screening by PCR and culture

Maternal sample						
Collection time	Antepartum		At the time of delivery		Before discharge	Postpartum one month
Collection site	Vaginal and rectal		Vaginal and rectal		Vaginal and rectal	Breast milk
Status	Culture	Sample A	Culture	Sample B	Sample C	Sample D
		PCR		PCR	PCR	PCR
Number of implementations	516	505	94	470	427	51
	98.3% (516/525)	96.2% (505/525)	17.9% (94/525)	89.5% (470/525)	81.3% (427/525)	9.71% (51/525)
Positive	83	94	11	78	63	6
	16.1% (83/516)	18.6% (94/505)	11.7% (11/94)	16.6% (78/470)	14.8% (63/427)	11.8% (6/51)
Negative	433	402	83	390	363	45
	83.9% (433/516)	79.6% (402/505)	88.3% (83/94)	83.0% (390/470)	85.0% (363/427)	88.2% (45/51)
inspection error	N/A	9	N/A	2	1	0
	N/A	1.78% (9/505)	N/A	0.426% (2/470)	0.234% (1/427)	0.00% (0/51)

Neonatal sample						
Collection time	At birth		Before discharge		One month-old	
Collection site	Oral	Rectal	Oral	Rectal	Oral	Rectal
Status	Sample P	Sample Q	Sample R	Sample S	Sample T	Sample U
	PCR	PCR	PCR	PCR	PCR	PCR
Number of implementations	466	467	319	319	391	392
	88.8% (466/525)	89.0% (467/525)	60.8% (319/525)	60.8% (319/525)	74.5% (391/525)	74.7% (392/525)
Positive	11	12	5	16	11	26
	2.36% (11/466)	2.57% (12/467)	1.57% (5/319)	5.02% (16/319)	2.81% (11/391)	6.63% (26/392)
Negative	453	453	311	299	370	357
	97.2% (453/466)	97.0% (453/467)	97.5% (311/319)	93.7% (299/319)	94.6% (370/391)	91.1% (357/392)
inspection error	2	2	3	4	10	9
	0.429% (2/466)	0.428% (2/467)	0.941% (3/319)	1.25% (4/319)	2.56% (10/391)	2.30% (9/392)

Both test results (culture and PCR) were available from 492 of the 525 participants antepartum and from 87 of the 525 participants at the onset of delivery. Considering the results of culture at the onset of delivery as true GBS colonization, the sensitivity, specificity, positive predictive value, and negative predictive value of the antepartum culture were 45.5%, 95.9%, 62.5%, and 92.1%, respectively (Table 5). Antepartum culture screening, which is the gold standard recommended by Japanese guidelines, exhibited lower sensitivity (45.5%), specificity (95.9%), negative predictive value (92.1%), and positive predictive value (62.5%). In contrast, the PCR test at the onset of delivery exhibited higher sensitivity (90.9%, *p* = 0.0221), specificity (98.6%, *p* = 0.311), negative predictive value (90.9%, *p* = 0.0599), and positive predictive value (98.6%, *p* = 0.134) than antepartum culture screening (Table 5).

**Table 5.** Performance of culture and PCR in antepartum and culture and PCR at onset delivery

	Antepartum		At the time of delivery		
	Vaginal and rectal		Vaginal and rectal		
	*n* = 84				
	Culture	PCR	Culture	PCR	n
	Negative	Negative	Negative	Negative	70
	**Positive**	**Positive**	**Positive**	**Positive**	5
	Negative	Negative	**Positive**	**Positive**	4
	Negative	**Positive**	**Positive**	**Positive**	1
	Negative	Negative	**Positive**	Negative	1
	**Positive**	**Positive**	Negative	Negative	2
	**Positive**	**Positive**	Negative	**Positive**	1
GBS detection rate (%)	9.52	10.7	13.1	13.1	84
Sensitivity (%)	45.5	54.5	N/A	90.9	
Specificity (%)	95.9	95.9	N/A	98.6	
Positive predictive value: PPV (%)	62.5	66.7	N/A	90.9	
Negative predictive value: NPV (%)	92.1	93.3	N/A	98.6	

Additionally, 84 participants had both GBS test results (culture and PCR) available at the antepartum and onset of delivery. Among these 84 participants, the concordance rate of culture results was 89.3% (75/84, 5 positive–positive and 70 negative–negative). Of the nine participants with discrepancies between the two results of culture tests, six changed from negative to positive, and three changed from positive to negative. In other words, among the 76 participants with a negative antepartum culture test, 7.89% (6/76) had a positive test result at the onset of delivery, and 37.5% (3/8) of participants with a positive antepartum culture test result had a negative test result at the onset of delivery.

These six participants completed delivery without IAP, and excessive IAP may have been performed in three participants. The time between antepartum GBS screening and delivery was not statistically different between cases of antepartum and onset-of-delivery GBS colonization discordance (16.8 ± 8.6 days) and concordance (19.1 ± 8.7 days, *p* = 0.378).

### Risk factors of mother-to-neonate GBS transmission

The number of available paired samples of both maternal and neonatal specimens was 468 out of 525. Mothers who tested positive for GBS by PCR at the time of delivery constituted 16.7% (78/468). Considering mothers with GBS positivity by PCR at the time of delivery, the detection rates of GBS in the neonatal rectum and/or oral cavity immediately after birth, in 4-day-old and 1-month-old neonates, were 15.4%, 11.5%, and 19.2%, respectively (Table 6). GBS was detected in neonates at 1 month old regardless of intrapartum antibiotic use (Table 6).

**Table 6.** GBS transmission from mother to infant.

			**GBS positive**		GBS negative		
			68	10	168	222	
			*n =* 78		*n* = 390		
			IAP	IAP	IAP	IAP	*n* (%)
			(+)	(−)	(+)	(−)	
Negative	Negative	Negative	49	5	163	210	427 (91.2)
Negative	Negative	**Positive**	7	0	4	3	14 (3.0)
Negative	**Positive**	**Positive**	2	1	1	4	8 (1.7)
Negative	**Positive**	Negative	2	0	0	2	4 (0.9)
**Positive**	**Positive**	**Positive**	2	2	0	1	5 (1.1)
**Positive**	**Positive**	Negative	0	0	0	0	0 (0.0)
**Positive**	Negative	Negative	6	1	0	2	9 (1.9)
**Positive**	Negative	**Positive**	0	1	0	0	1 (0.2)
**At birth**	**4 days old**	**30 days old**	**PCR at the time of delivery**				
**Neonates**			**Mothers**				

Additionally, 87.2% of PCR-detected GBS-positive mothers at the time of delivery (68/78) underwent intrapartum antibiotic treatment. Excluding 10 mothers who did not undergo PCR testing in the postpartum period, GBS was redetected at least on postpartum day 3 in approximately 51% of mothers with intrapartum antibiotic treatment (35/68). The GBS detection rate of nipple scrapings and breast milk samples from mothers who were GBS-positive at least once before delivery was 11.8% (6/51).

**Figure 2.**
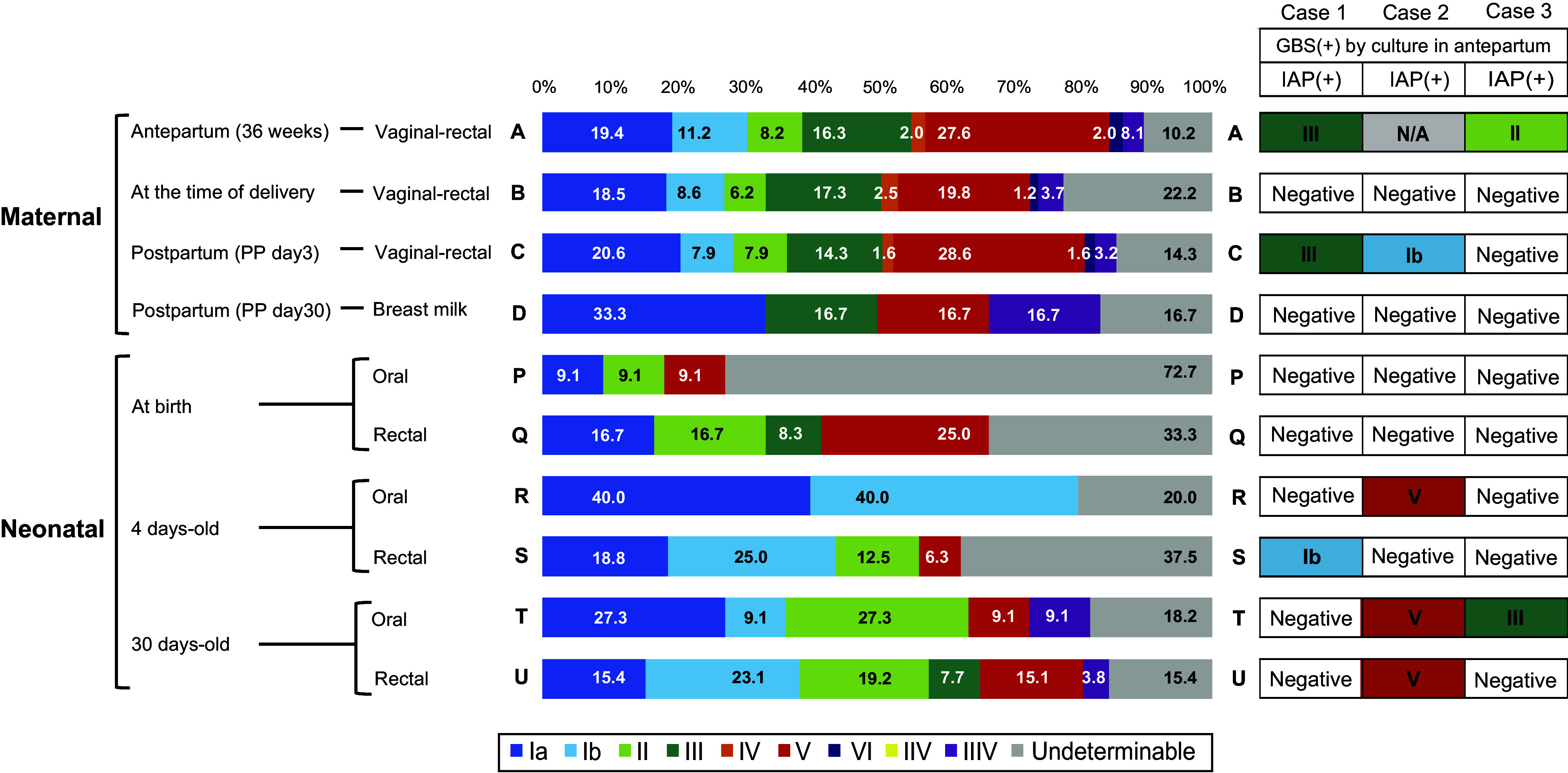
Comparison of the capsular types between maternal specimen and neonatal specimen. Each capsular type was expressed with a different color, and maternal GBS and neonatal GBS had different capsular types. There were 19 cases of both maternal and neonatal GBS positive cases. Of these, three cases (Cases 1–3) had different capsular type of isolated GBS between mothers and their neonate.

On the other hand, GBS was also detected in neonates from mothers GBS-negative before delivery (Table 6). In neonates from GBS-negative mothers before delivery, the detection rates of GBS immediately after birth, 4-day-old, and 1-month-old neonates were 0.77%, 2.1%, and 3.3%, respectively (Table 6). Although all these rates were significantly lower than those of mothers who were GBS-positive even once, the GBS detection rate of neonates at 1 month old was higher than immediately after birth, regardless of the detection method (Table 6). In neonates from never-GBS-positive mothers before delivery, no significant differences were observed in the family structure between GBS-positive and negative 1-month-old neonates. To examine the possibility of GBS transmission from individuals other than the mother, the capsular types were compared between the maternal and neonatal specimens (Figure 2). As a result, the ratio of the GBS capsular type was markedly different between the mother and neonates. In addition, there was similarity in the capsular type ratio based on the collection site. Samples A, B, and C were collected from the maternal vagina and rectum, while samples Q, S, and U were collected from the neonatal rectum, and samples P, R, and T were collected from the neonatal oral cavity. Thus, there was similarity among maternal vaginal-rectal specimens (samples A, B, and C), and there was also similarity, although lower, between maternal vaginal-rectal specimens and neonatal rectal specimens (samples Q, S, and U).

Regarding the ratio of the capsular type of neonatal rectal specimens at 1 month old, there was an increase in the ratio of types Ib and II. In the maternal vaginal-rectal specimens (samples A, B, and C), type V was the most common, followed by types Ia and III. Among these, types V, Ia, and III accounted for approximately half of the samples. The capsular type ratio of breast milk with nipple-scraping specimens (Sample D) was similar to that of the maternal rectal specimens; however, type Ia accounted for 30% of samples. Two different GBS capsular types were isolated simultaneously in 0.76% of cases (4/525): Ia and VIII, Ia and Ib, Ia and III, and Ib and III. In 495 participants with available maternal and neonatal paired samples, three cases of GBS capsule-type discordance between the mother and neonate were identified (Figure 2).

## Discussion

In this study, we verified the fluctuations in maternal GBS colonization from the antepartum to postnatal stages and the rate of transmission to neonates. We found that 3.5% of neonates born to GBS-negative mothers newly developed GBS colonization after 4 days of age, and the GBS capsular type distribution isolated from mothers and neonates was markedly different.

Although the lag between antepartum GBS screening and delivery was within 5 weeks, there was still a 10.7% discrepancy in GBS colonization between antepartum and onset delivery, as described in other published reports [7, 23, 24].

Of these results, 7.1% of cases changed from negative to positive, which might lead to a poor outcome, as IAP is not performed when antepartum negative results are found. In the GBS test at the time of delivery, one case each was culture-negative, PCR-positive, culture-positive, and PCR-negative (1/84, 1.1%). Antepartum GBS screening was culture-negative and PCR-positive. Antepartum GBS screening results were positive in both culture-positive and PCR-negative cases. To reduce false negatives as much as possible, an additional GBS test is performed at the time of delivery, and IAP is performed in GBS-positive cases. Thus, additional point-of-care testing at the time of delivery is important to evaluate maternal GBS infection. Similar to previous reports, our PCR detection test also had superior accuracy (sensitivity, 90.9%; specificity, 98.6%) compared to culture screening, even when using a GBS-selective culture medium at both intrapartum and delivery onset.

The American College of Obstetricians and Gynecologists (ACOG) guidelines also mention the usefulness of nucleic acid amplification tests (NAAT), including PCR, as alternatives to cultures [21]. However, to reduce the false negative rate, the ACOG suggests that the NAAT protocol should include an 18–24-h incubation step in the enrichment broth before performing the analysis [25–28]. Notably, our PCR detection method was performed by direct amplification without an enrichment cultivation step and was equivalent to culture-based screening. This indicates that if our PCR method can achieve the required speed, it may be able to meet the ACOG requirements.

However, even when based on accurate GBS detection, the prevention of GBS transmission by IAP is limited. In other words, IAP significantly prevented neonate transmission at birth (11.8% vs. 40.0%, *p* < 0.05), but there was no significant difference in GBS transmission rates at 4 and 30 days of age. Among the 453 neonates in whom GBS infection was not detected at birth, 26 (5.7%) became GBS-positive at 4 days old. Surprisingly, more than half of these 26 neonates (14/26) were born to mothers who tested negative for GBS at delivery. This suggests that regardless of the maternal GBS colonization status at birth, there is a 5.7% chance of acquiring GBS through horizontal transmission by 1 month old, indicating the potential for horizontal transmission in hospitals or households.

To evaluate potential bidirectional horizontal transmission routes, environmental factors, including family structure with infants and the presence of contact opportunities between the mother and infants, were examined. These environmental factors were not identified as risk factors for maternal GBS colonization during the prenatal period. A history of a GBS-positive result for a maternal culture test was identified as an independent risk factor. However, this result was based on self-reported data from the self-administered medical questionnaires. Therefore, there is a possibility that pregnant women may not accurately know their own GBS history, which represents a limitation of this analysis. If a history of positive GBS test is a risk factor, it is also crucial for pregnant women to correctly identify this history as a risk factor for mother-to-child infection transmission.

In addition, by comparing paired isolated GBS cases from mothers and their neonates, we found that the GBS capsular type distribution was markedly different between mothers and neonates.

These findings suggest that differences in fixation properties depend on the capsular type and the possibility of another GBS infection route besides vertical transmission. Breast milk may be a horizontal infection route [29–31]; in fact, 11.8% of prenatal GBS-positive mothers in this survey had GBS detected in their breast milk. Although there were no cases of neonatal GBS infection in this surveillance study, the finding of latent GBS carriages in GBS-positive pregnant women may be a route of LOD infection, for which IAP is ineffective. Even after IAP, GBS was detected in the vagina of 60.3% of GBS-positive mothers on postpartum day 3, suggesting that postpartum horizontal infection may have occurred in the mother.

As there are various potential horizontal routes of infection, measures against horizontal infections are necessary.

The GBS vaccine is expected to be a method for preventing LOD. The WHO has prioritized the development of GBS vaccines, and clinical trials are underway in various countries. The cost-effectiveness of the GBS vaccine is estimated to be high even in Japan [20]. In the development of GBS vaccines, a combination of capsular types is important for prevention. However, large-scale nationwide surveys of capsular type distribution in GBS-colonizing pregnant Japanese women are rare. In this study, we showed the capsular type distribution of 525 mother-neonate pairs, which represents the largest such report in Japan. Japanese institutes have reported a significant change in capsular type distribution over the past 20 years [32–34]. In the late 1990s, capsular types VIII and VI, which have weak pathogens, were predominant, accounting for more than 50% of the colonizing strains [33, 35]. The detection of capsular types Ia, III, and V, which have strong pathogens, increased between 2000 and 2010 [33, 36]. The recent maternal capsular type distribution in Japan is consistent with the globally predominant Ia, Ib, III, and V capsular types [37]. In our survey, capsular Ia, Ib, III, and V with high virulence were predominant, accounting for more than 50% of cases. This dynamic shift in distribution may explain the increased incidence of neonatal GBS infections. Moreover, the simultaneous detection of the two different capsular types indicated the possibility of capturing the colonized capsular type shift.

One of the vaccines in development (GBS6) [38], which includes six dominant GBS capsular types, is expected to cover more than 90% of neonatal invasive strains in Japan [33, 39]. In any case, prevention by IAP or vaccines is incomplete, and as long as a constant horizontal transmission route exists, the potential risk of neonatal GBS infection will not disappear.

Several limitations remain in this study. First, this survey was not a nationwide study; it may not reflect regional differences in incidence rates. Second, there were no cases of neonatal GBS infection. Third, although we could reveal the timing of GBS colonization the route of infection of invasive GBS disease is still unknown.

In conclusion, precautionary measures are essential. In addition, a highly accurate rapid diagnosis would be extremely useful not only for antepartum mothers but also for early therapeutic intervention for neonates who unfortunately develop GBS infection. A rapid and highly accurate molecular GBS detection method that can be easily performed within 24 h without being limited to certain facilities could become a breakthrough in the diagnosis and treatment of neonatal GBS infections.

## Supporting information

Yoshida et al. supplementary material Yoshida et al. supplementary material

## Data Availability

The datasets used and/or analyzed during the current study are available from the corresponding author on reasonable request.

## Supplementary material

For supplementary material accompanying this paper visit http://doi.org/10.1017/S0950268824001560. click here to view supplementary material
