# Prospective study of peripartum group B streptococcus colonization in Japanese mothers and neonates – ERRATUM

**DOI:** 10.1017/S0950268825000020

**Published:** 2025-01-27

**Authors:** Emiko Yoshida, Jun Takeda, Yojiro Maruyama, Naoko Suga, Satoru Takeda, Hajime Arai, Atsuo Itakura, Shintaro Makino

**Affiliations:** 1 Department of Obstetrics and Gynecology, Juntendo University, Tokyo, Japan; 2 Diagnostics and Therapeutics of Intractable Diseases, Intractable Disease Research Center, Graduate School of Medicine, Juntendo University, Tokyo, Japan; 3 Department of Obstetrics and Gynecology, Juntendo University Nerima Hospital, Tokyo, Japan; 4 Department of Obstetrics and Gynecology, Juntendo University Urayasu Hospital, Chiba, Japan; 5 Aiiku Research Institute for Maternal, Child Health, and Welfare, Tokyo, Japan; 6 Department of Neurosurgery, Juntendo University, Tokyo, Japan

When this article was originally published in Epidemiology and Infection it contained an error in the affiliation for the author Satoru Takeda. This was due to a comment erroneously being inserted into the affiliation during production. This has now been updated and corrected.

The publisher apologises for this error.
