# Supplementary material for: Prospective study of peripartum group B streptococcus colonization in Japanese mothers and neonates
Source: Epidemiol Infect. 2025 Jan 6;153:e1. doi: 10.1017/S0950268824001560 (PMC11704933; doi:10.1017/S0950268824001560)
Supplement: Yoshida et al. supplementary material [file S0950268824001560sup001.docx]

**Epidemiology and Infection**

**Prospective study of peripartum Group B Streptococcus colonization in Japanese mothers and neonates**

Emiko Yoshida^1,2^, Jun Takeda^1^, Yojiro Maruyama^3^, Naoko Suga^4^, Satoru Takeda^1,5^, Hajime Arai^6^, Atsuo Itakura^1^, Shintaro Makino4*

^1^ Department of Obstetrics and Gynecology, Juntendo University, Tokyo, Japan

^2^ Diagnostics and Therapeutics of Intractable Diseases, Intractable Disease Research Center, Graduate School of Medicine, Juntendo University, Tokyo, Japan

^3^ Department of Obstetrics and Gynecology, Juntendo University Nerima Hospital, Tokyo, Japan

^4^ Department of Obstetrics and Gynecology, Juntendo University Urayasu Hospital, Chiba, Japan

^5^ Aiiku Research Institute for Maternal, Child Health and Welfare, Tokyo, Japan

^6^ Department of Neurosurgery, Juntendo University, Tokyo, Japan

*Corresponding author

**Supplementary Materials**

**Supplementary Table S1: Result of self-administered medical questionnaire**

|  | **Nulliparous** | n=332 |  | **Primiparous** | n=193 |
| --- | --- | --- | --- | --- | --- |
| **Variable** | **n** | **(%)** |  | **n** | **(%)** |
| **Number of valid responses** | 328 | 98.8 |  | 190 | 98.4 |
| **Experience of GBS *** | | | | | |
| Never experience | 318 | 95.8 |  | 170 | 88.1 |
| Diagnosed with GBS | 10 | 3.0 |  | 19 | 9.8 |
| when not pregnant | 1 | 0.3 |  | 0 | 0.0 |
| when pregnant | 9 | 2.7 |  | 19 | 9.8 |
| Unanswered | 0 | 0.0 |  | 1 | 0.5 |
| **History of use of menstrual control drugs *** |  |  |  |  |  |
| Never experience | 165 | 49.7 |  | 117 | 60.6 |
| Yes | 163 | 49.1 |  | 73 | 37.8 |
| Low dose Estrogen Progestin (with overlap) | 139 | 41.9 |  | 63 | 32.6 |
| Progestin (with overlap) | 33 | 9.9 |  | 8 | 4.1 |
| gonadotropin-releasing hormone (GnRH)   antagonists/analogs (with overlap) | 10 | 3.0 |  | 2 | 1.0 |
| intrauterine device (with overlap) | 3 | 0.9 |  | 0 | 0.0 |
| others/details unknown (with overlap) | 10 | 3.0 |  | 4 | 2.1 |
| Unanswered | 0 | 0.0 |  | 1 | 0.5 |
| **family living together *** | | | | | |
| Over 18-year-old adults only | 326 | 98.2 |  | 6 | 3.1 |
| with children | 0 | 0.0 |  | 184 | 95.3 |
| 7-18 year-old children | 0 | 0.0 |  | 49 | 25.4 |
| infants with/without 7-18 year-old children | 0 | 0.0 |  | 135 | 69.9 |
| Unanswered | 2 | 0.6 |  | 0 | 0.0 |
| **Opportunities for contact with infants within the last year *** | |  |  |  |  |
| Never | 250 | 75.3 |  | 34 | 17.6 |
| Yes | 78 | 23.5 |  | 156 | 80.8 |
| up to one year ago / before pregnancy | 13 | 3.9 |  | 11 | 5.7 |
| also after pregnancy / even now | 65 | 19.6 |  | 145 | 75.1 |
| Unanswered | 0 | 0.0 |  | 0 | 0.0 |

Note

*: The denominator is the total number of valid responses (Nulliparous 328, Primiparous 190).

**Staffs of Juntendo University hospital, Urayasu Hospital and Nerima Hospital**

Doctors, nurses and staffs of Juntendo Hospital:

Yuka Yamamoto, Anna Sato, Yuri Ishida, Nana Matsuzawa, Eri Kitamura, Rie Seyama, Hitomi Ando, Kyoko Namimaysu, Shun Masaoka, Nami Tamura, Eimi Seo, Yusen Sugimura, Haruka Mieda, Tsukasa Yoshida, Taihei Yamada, Yukiko Okada, Kazunari Fujino, Takashi Hirayama, Keisuke Murakami, Atsuko Yamada, Yu Kawasaki, Mutsumi Kobayashi, Shiori Takeuchi, Kengo Hiranuma, Sumire Ishii, Yasuho Yanagihara, Natsuko Yamji, Misato Kawata, Koji Ijichi, Mayu Sugawara, Saori Otsuka, Yuko Isozaki, Kyoko Sakurai, Lisa Nishimura, Kanako Iwai, Kamata Mitsuko, Mineko Furukawa, Makiko Sagawa, Midori Iwamitsu, Manami Ajioka, Maya Suzuki, Azusa Miyahara, Sae Kasai, Mayu Takigawa, Nao Yoshiki, Mako Morinaga, Marina Oshima, Chihiro Uchiyama, Chikako Sakai, Momoka Iwai, Ayumi Tobita, Sari Hashiba, Yuki Haginiwa, Rino Fujita, Ayano Yamagishi, Lise Toyama, Yoko Ariake, Sanae Kurihara, Chihiro Shirato, Chiharu Nishikawa, Momoe Nakagami, Nahoko Ito, Mina Horikiri, Yumi Kobayashi, Sayaka Ikeda, Asami Matsuo, Haruka Sato, Arisa Terashima, Ayu Goto, Ayaka Kurata, Misato Masuko, Mayumi Iwata, Emiko Idehara, Akiko Sekiguchi, Yumiko Abe, Rei Ishida, Nagisa Yoshimura, Mami Kishima, Yu Hokazono, Aya Egashira, Mitsuyo Kosuge, and Naoko Yumoto

Doctors and nurses of Juntendo Urayasu Hospital: Akari Koizumi, Ai Takamizu, Saki Ito, Junya Akiba, Masafumi Segawa, Norikazu Ueki, Yuki Kitajima and Mariko Aoki

Doctors and nurses of Juntendo Nerima Hospital: Yuka Kido, Riho Mera, Sakae Terao, Hiroko Kawai, Utako Negoro, Miki Sasaki, and Ena Sekiguchi
